# Long-term trends in blood pressure and hypertension in Russia: an analysis of data from 14 health surveys conducted in 1975–2017

**DOI:** 10.1186/s12889-021-12320-4

**Published:** 2021-12-07

**Authors:** Elena Churilova, Vladimir M. Shkolnikov, Svetlana A. Shalnova, Alexander V. Kudryavtsev, Sofia Malyutina, Odd Nilssen, Tiina Laatikainen, David A. Leon

**Affiliations:** 1 National Research University Higher School of Economics, Bolshoy Trekhsvyatitelskiy pereulok 3, Moscow, Russian Federation 109038; 2 Max Planck Institute for Demographic Research, Konrad-Zuse-Str. 1, 18057 Rostock, Germany; 3 National Medical Research Centre for Therapy and Preventive Medicine, Petroverigskiy pereulok 10, Moscow, Russian Federation 101990; 4 Northern State Medical University, Troitsky Avenue 51, Arkhangelsk, Russian Federation 163069; 5 UiT The Arctic University of Norway, 9037 Tromsø, Norway; 6 Research Institute of Internal and Preventive Medicine, Branch of Institute of Cytology and Genetics, Siberian Branch of the Russian Academy of Sciences, B. Bogatkova str. 175/1, Novosibirsk, Russian Federation 630089; 7 Novosibirsk State Medical University, Russian Ministry of Health, Krasny pr. 52, Novosibirsk, Russian Federation 6300091; 8 Finnish Institute for Health and Welfare, P.O. Box 30, FI-00271 Helsinki, Finland; 9 Institute of Public Health and Clinical Nutrition, University of Eastern Finland, P.O. Box 1627, FI-70211 Kuopio, Finland; 10 London School of Hygiene & Tropical Medicine, London, WC1E 7HT UK

**Keywords:** Blood pressure, Hypertension, Russia, International differences, Meta-analysis

## Abstract

**Background:**

Hypertension is recognized as an important contributor to high cardiovascular mortality in Russia. A comprehensive analysis of data from Russian studies that measured blood pressure in population-based samples has not been previously undertaken. This study aims to identify trends and patterns in mean blood pressure and the prevalence of hypertension in Russia over the most recent 40 years.

**Methods:**

We obtained anonymized individual records of blood pressure measurements from 14 surveys conducted in Russia in 1975–2017 relating to a total of 137,687 individuals. For comparative purposes we obtained equivalent data from 4 surveys in the USA and England for 23,864 individuals. A meta-regression on aggregated data adjusted for education was undertaken to estimate time trends in mean systolic and diastolic blood pressure, the prevalence of elevated blood pressure (> 140/90 mmHg), and hypertension (defined as elevated blood pressure and/or the use of blood pressure-lowering) medication. A meta-analysis of pooled individual-level data was used to assess male-female differences in blood pressure and hypertension.

**Results:**

During the period 1975–2017 mean blood pressure, the prevalence of elevated blood pressure and hypertension remained stable among Russian men. Among Russian women, mean systolic blood pressure decreased at an annual rate of 0.25 mmHg (*p* < 0.1) at age 35–54 years and by 0.8 mmHg (*p* < 0.01) at ages 55 and over. The prevalence of elevated blood pressure also decreased by 0.8% per year (*p* < 0.01), but the prevalence of hypertension remained stable. Mean blood pressure and prevalence of hypertension were higher in Russia compared to the USA and England at all ages and for both sexes.

**Conclusions:**

In contrast to the generally observed downward trend in elevated blood pressure in many other countries, levels in Russia have changed little over the past 40 years, although there are some positive trends among women. Improved strategies to bring down the high levels of mean blood pressure and hypertension in Russia compared to countries such as England and the USA are important to further reduce the high burden of CVD in Russia.

**Supplementary Information:**

The online version contains supplementary material available at 10.1186/s12889-021-12320-4.

## Background

Blood pressure is a strong predictor of cardiovascular mortality [1] and elevated levels are a leading determinant of premature cardiovascular disease (CVD) globally [2, 3]. Russia has had one of the highest cardiovascular disease mortality rates in the world, even though since 2005 rates have been declining [4]. A Global Burden of Diseases (GBD) analysis on Russia concluded that in 2016 high systolic blood pressure was responsible for over 33% of all deaths [5].

During the last 5 years, several global studies have been published in which mean blood pressure and the prevalence of hypertension have been compared between countries and regions. Based on pooled data from 1479 studies the NCD Risk Factor Collaboration (NCD-RisC) [6] found that systolic blood pressure (SBP) and diastolic blood pressure (DBP) were high in countries of Central and Eastern Europe (CEE) relative to other regions, although levels had reduced among women of the region over the past 4 decades. An analysis of international variation in hypertension in 2010 (based on World Health Organization (WHO) Study on global AGEing and adult health (SAGE) data) found Russia to have one of the highest prevalences [7]. An analysis of data from the GBD found that the age-standardized prevalence of elevated blood pressure was particularly high in Russia, and other former Soviet countries, compared to those of Western Europe: 29–31% in Russia, Ukraine, Belarus, and Estonia vs. 15.2% in the UK, 20% in France and Denmark [8]. These large data-synthesis analyses were based on studies from a diverse range of surveys from across the world that the project teams had been able to assemble.

In Russia since the late 1970s, a number of population-based health surveys have been conducted that measured blood pressure and collected information on the use of anti-hypertensive medications. The earliest estimates of hypertension prevalence in Russia were from the collaborative USSR-USA Lipid Research Clinics Prevalence Study (LRC) conducted in 1975–77 and 1982–84 [9–11]. Subsequent rounds of the LRC, as well as the World Health Organization MONItoring of Trends and Determinants in Cardiovascular disease (MONICA) were conducted in the 1990s and the early 2000s [12]. In the 2000s and the 2010s, several other large population-based and multi-regional surveys have been carried out [13–17]. Since 2012 the Russian Ministry of Health has funded several rounds of the ESSE survey focused on cardiovascular disease in different regions of Russia [13], although unlike the National Health and Nutrition Examination Survey (NHANES) in the USA or the Health Survey for England (HSE) these were not based on nationally representative population samples. In addition, there have been studies conducted in specific cities or regions of the country [18–23]. To date, no attempt has been made to bring these data together to provide a systematic analysis of trends in mean systolic and diastolic blood pressure, prevalence of elevated blood pressure, and hypertension by age and sex in Russia. In the present study, we undertook such a comprehensive assessment based on datasets from Russian surveys conducted over the past 40 years.

## Methods

We obtained from investigators data from studies that had measured blood pressure in population-based samples in Russia with adequate documentation of their design and protocols. To be included studies had to have aimed at being representative of their target population, defined either as the entire country, one or several regions, or defined geographic areas. Although the precise designs and settings varied among studies, our analysis aims at identifying any common patterns that may reflect true underlying characteristics in the general population of Russia. Studies were identified based on our extensive knowledge of Russian population-based research. We supplemented this in January-February 2019 with a Google search for “hypertension AND Russian population” and “blood pressure AND Russian population”. This did not yield any studies that we were not already aware of. We approached investigators of all the studies we considered to be eligible for inclusion. As we discuss later in the paper only one study refused to provide their data.

### Data sets

Our analysis is based on individual blood pressure measurements from 14 independent cross-sectional surveys conducted in Russia that jointly included a total of 137,687 participants (Supplementary Table S1). Among these surveys only the WHO 2007–2010 SAGE aimed at being representative of the general population of the whole country. Other epidemiological studies were restricted to one or more sub-national areas. The sample sizes varied from 483 in Pitkäranta (conducted in 2007) to 31,949 in the Monitoring of the Arterial Hypertension (Monitoring AH, 2005–2008). Half of the surveys cover the range of ages 18 years and older. However, SAGE and Stress, Ageing and Health Study (SAHR) included only those aged 55+ years. The Pitkäranta studies and the Izhevsk Family Study 2 (IFS) include only individuals at working ages 25–64 years, while the Know Your Heart (KYH) study was restricted to those aged 35–69 years. To put our results in the international context, we obtained measurements on 23,864 participants from the 2009 and 2016 from the Health Survey for Englad (HSE) [24, 25] and 2007–2008 and 2015–2016 rounds of the the US NHANES [26, 27]. Characteristics of data sets and populations analyzed are provided in Supplementary Table S1.

### Ethics and consent

This study involved the secondary analysis of a small number of variables in anonymized datasets from a variety of sources. All procedures in surveys included in analysis were performed in accordance with relevant guidelines. No ethical approval is needed to access any data used in this study.

### Definitions

Elevated blood pressure was defined as SBP of 140 mmHg or higher or DBP of 90 mmHg or higher. Where available we used the mean of two or more blood pressure measurements as appropriate. More details on the number of blood pressure measurements and the type of equipment used in each study are provided in Supplementary Table S2.

We used self-reported use of blood pressure-lowering drugs. This information was obtained in answer to a variety of questions: “Have you been taking any medications or other treatment for elevated blood pressure during the last 2 weeks?” (MONICA, Monitoring AH, SAHR, SAGE and during 1 week for LRC), “Have you been taking any medications lowering blood pressure? (Arkhangelsk study, ESSE), “Do you take prescribed drugs to control your blood pressure?” (IFS2). In SAGE and NHANES the question about blood pressure medication was asked to those who had been informed by a doctor about blood pressure elevation, in other studies to all respondents. For 4/14 of the studies information about the precise question asked about medication was not available.

In all studies, hypertension was defined as measured elevated blood pressure and/or the use of blood pressure-lowering medication.

In each study, we aggregated education level reported by respondent into three categories: high, middle, and low education. High education corresponds to a university degree or another type of higher education degree. Middle education corresponds to completed secondary education or vocational secondary education. Low education means less than completed secondary education.

### Statistical analyses

Our analysis started with an inspection of the age profiles (10-year intervals from 35 to 44 to 75+) of the mean SBP and DBP and the prevalence of elevated blood pressure and hypertension (Supplementary Tables S3, S4, S5 and S6). We found that education tended to be inversely associated with mean and elevated blood pressure that also agrees with earlier study [28]. To remove potential confounding effects of differences in the educational composition of the different study populations, in part generated by selection bias, we standardized all estimates to the educational structure reported at the 2002 All-Russia Census [29].

To examine temporal trends we looked at whether mean levels of blood pressure and hypertension tended to be lower in later surveys compared to earlier surveys. These analyses were stratified by sex and age (groups 35–54 and 55+ years) with cutoffs chosen so that we had adequate numbers in each age group on which to estimate means and proportions. The study-specific outcomes (mean SBP, mean DBP, prevalence of elevated blood pressure, prevalence of hypertension, prevalence of treated patients among hypertensives) were regressed on the central year of each survey with adjustment for mean age and the share of high education using a random-effects meta-regression model (metareg command in Stata) [30] on study-level summary data.

To investigate whether male-female differences in the blood pressure outcomes changed over time we compared the magnitude of the sex difference between and within studies according to central year of survey. We used *ordinary least squares (*OLS) meta-analysis regression (*ipdmetan* command in Stata) [31] on individual-level data with the “forest plot” option to estimate the male-female differences in mean systolic and diastolic blood pressure adjusting for education and age. An equivalent set of logistic meta-analysis models were used to estimate differences between studies in the male-female odds ratio for elevated blood pressure and hypertension. In order to deal with the fact that studies varied considerably in the age range of subjects they covered, these analyses were restricted to the age range 55–64 years as this age group was the one where data existed for all (14) Russian studies.

 All tabulations and regression models were implemented in Stata 14 [32].

## Results

### Mean systolic blood pressure

**Fig. 1:**
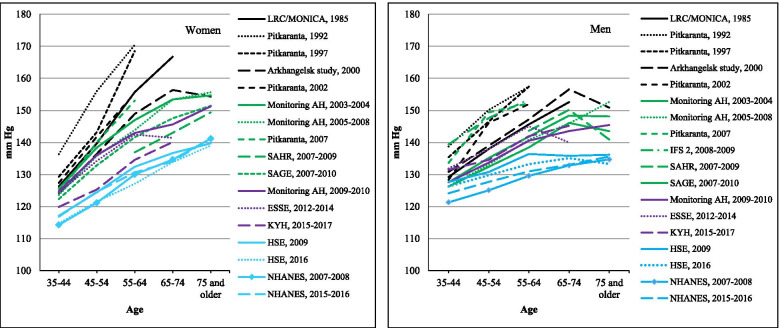
Age-specific mean SBP (mm Hg) in Russian surveys compared to HSE and NHANES. Notes: For Russian surveys, averages are standardized by education. For more details, see the Methods section

Figure 1 shows the education adjusted mean SBP values for men and women by age in each study. As expected, in all studies mean SBP tended to increase with age. All means from the Russian studies were higher than those in NHANES (USA) and HSE (England), with the exception of men in the youngest age groups. In general, the difference between the mean SBP in the Russian and the comparator Western surveys increased with age.

In both women and men, the highest SBP means in Russia tended to be found in the earliest studies conducted up until the early 2000s. Among women, the lowest values were found in the most recent study (KYH, 2015–17).

The trends over time for blood pressure means by central year of study were examined using meta-regression stratified by sex and age group 35–54 and 55+ years (Table 1). The results show a statistically significant decline over time in the mean SBP both among women aged 55 years and older of 0.8 mmHg per year (*p* < 0.01) and a marginally significant decline of 0.25 mmHg per year in the younger age group 35–54 (*p* < 0.1). There was little evidence for a decline in either age group among men.

**Table 1:** Trends over time^a^ in the blood pressure indicators by sex within age groups 35–54 and 55 years and older

Population group (sex x age)	Number of surveys in analysis	Slope (annual change) and 95% CI	*P*
Mean SBP (mm Hg) per year			
Women, 35–54	12	−0.252 (− 0.535, 0.031)	0.074
Women, 55+	13	**−0.786 (− 1.156, − 0.415)**	**0.001**
Men, 35–54	13	−0.087 (− 0.517, 0.343)	0.658
Men, 55+	14	−0.235 (− 0.615, 0.145)	0.199
Mean DBP (mm Hg) per year			
Women, 35–54	12	−0.157 (− 0.389, 0.075)	0.158
Women, 55+	13	−0.127 (− 0.315, 0.061)	0.161
Men, 35–54	13	−0.078 (− 0.336, 0.181)	0.515
Men, 55+	14	−0.005 (− 0.131, 0.121)	0.934
Prevalence of elevated blood pressure (%) per year			
Women, 35– 54	12	−0.425 (0.962, 0.111)	0.105
Women, 55+	13	**−0.785 (−1.223, − 0.348)**	**0.003**
Men, 35–54	13	−0.359 (−1.211, 0.492	0.365
Men, 55+	14	−0.217 (− 0.580, 0.145)	0.211
Prevalence of hypertension (%) per year			
Women, 35–54	12	−0.123 (− 0.520, 0.275)	0.497
Women, 55+	13	−0.073 (− 0.195, 0.048)	0.206
Men, 35–54	13	−0.089 (− 0.717, 0.537)	0.753
Men, 55+	14	0.221 (−0.119, 0.561)	0.179
Prevalence of anti-hypertensive medication use among hypertensives (%) per year			
Women, 35–54	12	**1.604 (0.054, 3.155)**	**0.044**
Women, 55+	13	**1.780 (0.760, 2.800)**	**0.003**
Men, 35–54	13	**1.532 (0.184, 2.881)**	**0.030**
Men, 55+	14	**1.889 (0.425, 3.352)**	**0.017**

^a^Results of meta-regression on data from 14 Russian surveys. Regression models are controlled for education and age. See the Methods section for more details

### Mean diastolic blood pressure

**Fig. 2:**
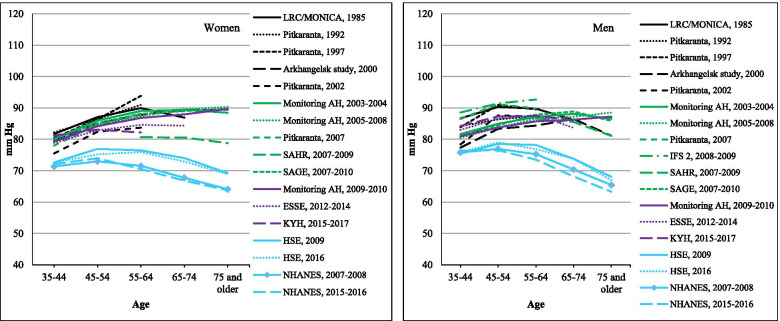
Age-specific mean DBP (mm Hg) in Russian surveys compared to HSE and NHANES. Notes: For Russian surveys, averages are standardized by education. For more details, see the Methods section

Figure 2 shows the education adjusted mean DBP values for men and women by age in each study. The range of means in the Russian studies at all ages was much narrower than seen for SBP: nearly all values lie in the range 80–90 mmHg. In most Russian studies the DBP means increased slightly with age or level off at age 65–74 years. In contrast, the NHANES and HSE surveys showed marked declines from age 55–64 years onwards. This resulted in a clear widening of DBP differences with age between the Russian means and those in the comparator countries.

Unlike SBP there was no obvious trend of DBP with year of survey. The meta-regression results (Table 1) confirm that there was little evidence of any trend over time in mean DBP for either men or women.

### Prevalence of elevated blood pressure

**Fig. 3:**
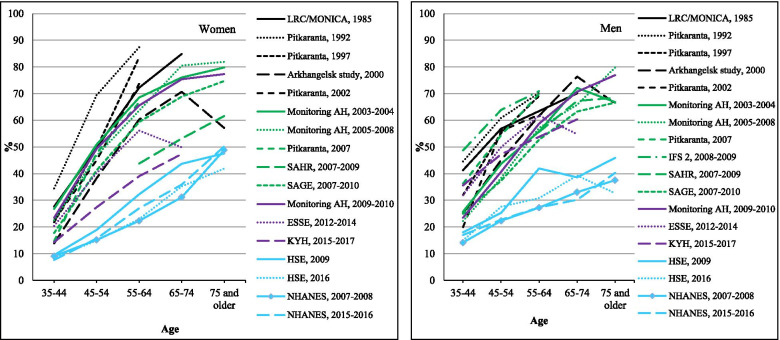
Age-specific prevalence of elevated blood pressure in Russian surveys compared to HSE and NHANES. Notes: For Russian surveys, averages are standardized by education. For more details, see the Methods section

Figure 3 shows the education-adjusted prevalence of elevated blood pressure (> = 140/90) for men and women by age in each study. In all studies, there was a clear tendency for prevalence to increase steeply with age. This increase was generally steeper for the Russian studies than for the comparator studies in the USA and England, resulting in a wider international gap at older than younger ages. The exception to this was the KYH (2015–17) study where the prevalences at every age in women were only slightly higher than those seen for the NHANES (USA) and HSE (England).

Among the Russian studies at any age, the highest prevalences were generally found in the earliest studies, although men in the IFS2 study (2008–9) had the highest levels. Among women, the lowest prevalence of elevated blood pressure was for the most recent survey (KYH 2015–17). The meta-regressions (Table 1) show that there was a downward trend with time in the prevalence of elevated blood pressure in the Russian studies in younger and older men and women, although this was only significant for women aged 55+ years.

### Prevalence of hypertension

**Fig. 4:**
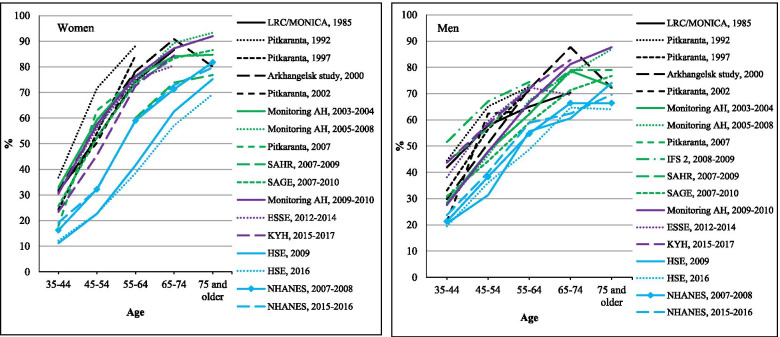
Age-specific prevalence of hypertension in Russian surveys compared to HSE and NHANES. Notes: For Russian surveys, percentages are standardized by education. For more details, see the Methods section

Figure 4 shows the age-specific education adjusted prevalence of hypertension in each study. Among men at every age, the NHANES and HSE prevalences were lower than seen in any Russian study. For women prevalences were much higher at each age in the Russian studies compared to NHANES or HSE, although it is intriguing to note that NHANES levels lie almost exactly between those of the Russian studies and the English (HSE) estimates.

For women, at any given age the Russian studies all showed very similar prevalences although once again the most recent study (KYH 2015–17) had the lowest prevalences. In contrast, there was a more heterogeneous pattern among men across the different Russian studies. The two most recent studies (ESSE 2012–2014 and KYH 2015–2017) showed slightly higher prevalences in middle age than those observed a decade earlier. Consistent with the impression from Fig. 4 there was no evidence from the meta-regression results (Table 1) for a change over time in prevalence in hypertension in the Russian studies for either men or women.

The numeric values underlying Figs. 1, 2, 3 and 4 are given in Supplementary Tables S3 to S6.

### Changes in anti-hypertensive medication

We analyzed the time trend in the percentage of people with hypertension who reported use of anti-hypertensive medications. Our results (Table 1) show that there was a significant upward trend with time in the prevalence of blood pressure medications use among Russian hypertensive. The results show a statistically significant increase in blood pressure medications use of 1.6% (*p* < 0.05) per year among women with hypertension aged 35–54 years and of 1.8% (*p* < 0.01) per year among hypertensive women aged 55+. Similarly among men, anti-hypertensive medications use also significantly increased by 1.5% (*p* < 0.05) at age 35–54 years and by 1.9% (*p* < 0.05) among those aged 55+ years.

### Gender differences

We have already noted that there are some differences between men and women in levels and trends. The majority of studies included participants in the age range 55–64 years, allowing us to analyze gender differences in blood pressure dynamics holding age constant. The results are shown in Supplmentary Figs. S1, S2, S3 and S4. During the 1980s and the 1990s, mean SBP was lower in men than women. However, mean SBP in women started decreasing in the 2000s, which resulted in a reduction in the SBP gender gap. The surveys conducted in the 2010s show SBP in men to be higher than in women for the first time (Supplementary Fig. S1). Similar gender-specific temporal changes were found in mean DBP and in the elevated blood pressure (Supplementary Figs. S2 and S3). The male-female differences in the prevalence of hypertension show female excess in the 1980s, the 1990s, and the 2000s (Supplementary Fig. S4). However, in the 2010s this gender difference in hypertension prevalence among 55–64 years-old disappeared.

## Discussion

Our analyses are the most detailed examination to date of all key dimensions of blood pressure and hypertension in Russia spanning over 40 years from 1975 to 2017. Our clearest finding is that mean blood pressures and the prevalence of elevated blood pressure and hypertension in the Russian surveys are higher than in the comparator surveys from the USA and England even in the most recent years. This is consistent with the continued excess of cardiovascular disease mortality in Russia compared to Western countries and underlines how important raised blood pressure is likely to be as a key driver of the very high burden of cardiovascular disease in Russia.

A key aims of this study has been to examine trends over time in Russia. Our results provide some evidence for improvements in mean blood pressure and prevalence of elevated blood pressure from the earliest to the most recent surveys. This evidence is strongest for women aged 55 years and older, with less sizeable and convincing trends apparent for men. This is broadly consistent with the conclusion of the NCD-RisC analysis [6] that reported that there was a tendency of mean blood pressures to decrease among women from the former communist countries of Eastern Europe.

Given these trends in mean and elevated blood pressures, it is striking that we found almost no evidence in either men or women of changes over time in the prevalence of hypertension per se defined in terms of elevated blood pressure (140/90) and/or taking antihypertensive medication. This might be explained by increases over time in the use of anti-hypertensives in Russia that were particularly pronounced among women. The higher the proportion of the population taking anti-hypertensive medication the higher the nominal prevalence of hypertension regardless of whether this translates into an impact on mean or elevated blood pressure. Certainly, even in the most recent survey, there are high rates of uncontrolled hypertension especially among men [33].

The decrease in mean blood pressure among women led to the initial gender difference in the prevalence of elevated blood pressure with women having the highest levels was reversed from the 2010s. This positive change among women combined with the growing use of medications among them may indicate more effective hypertension control than among men.

Our most unexpected finding concerned contrasts between mean SBP and mean DBP. Firstly, diastolic blood pressures for both men and women showed less variation across the Russian surveys than systolic blood pressure. Moreover, the gap between the Russian studies and those in England and the US was larger and more pronounced for DBP than SBP. Secondly, there were striking differences in the relationship of age to mean DBP in the Russian compared to the US and England. Whereas in the US and English surveys mean DBP showed a clear decline from the fifth decade of age, none of the Russian surveys included in analysis showed this effect. Instead, most of the Russian surveys showed either continued increases with age or a plateauing or small decline in DBP from around age 50 years. This is consistent with the small longitudinal declines seen in DBP in the Russian HAPIEE study [34].

In contrast to what we have noted for Russia, there is abundant other evidence of a pronounced decline in DBP after the age of 50 years in Western high-income countries both in cross-sectional and in longitudinal studies [35]. One explanation for this decline with age is that the process of vascular aging [36] involves increases in arterial stiffness [37]. Reduction in the elasticity of the aorta results in reduced blood pressure in diastole. However, it has been postulated that it is only from around 50 years of age that the downward influence on DBP overcomes the general upward tendency on both SBP and DBP to increase due to increases in peripheral vascular resistance with age [37]. Could it, therefore, be that in Russia at a population level there may be a steeper increase in the influence of peripheral vascular resistance with age than is seen in countries such as those in Western Europe and North America? This is consistent with the fact that our analyses show a steeper net increase in SBP with age than observed in the comparator national surveys from England and the USA.

One of the important influences on blood pressure levels in a population is diet especially salt intake [38]. It is out of the scope of this paper to consider how far the blood pressure trends and patterns may be explained by salt intake and other aspects of primary prevention. However, it should be noted that the failure to show declines in mean blood pressure other than among older women in Russia suggests that any attempts at the primary prevention of hypertension have not been effective.

The inevitable weakness of our study, and indeed of all other parallel data syntheses including the Global Burden of Disease and NCD-RisC [6], is that the studies we have been able to include use a range of designs, sampling frames, and sample sizes. Moreover, we and others depend upon the willingness of other researchers to share their data. In our case we obtained data from all of the studies that we classed as eligible with one exception [34].

The optimal approach to study population trends in a parameter such as blood pressure would be to have data from a single study of a consistently defined geographic location sampled at random which used a consistent recruitment and measurement methodology at successive time points. This is the basic design of the Health Survey for England and the US NHANES we used as international comparators in this study. However, in Russia, there are no equivalent repeated large-scale nationally representative surveys. The Russian Longitudinal Monitoring Survey (RLMS) which we have previously used to study trends in smoking [39] has not measured blood pressure. Moreover, the AH Monitoring studies conducted in 2003–05, 2005–08, and 2009–10, that are included in our analysis, differ from each other in terms of the regions participating in these studies. In this situation, we have therefore had to use the best available alternative of synthesizing data from different studies conducted over a span of years.

Some of the studies we used recruited participants via health care facilities while others used population lists as a basis for sampling. Only one of the Russian studies explicitly attempted to draw a nationally representative sample, while many of the studies were restricted to one or two regions or cities. This diversity in survey location, design, and size will almost certainly have introduced sources of heterogeneity that will make temporal trends challenging to identify with certainty. To minimize some of the extraneous sources of variation, we used methods of meta-analysis that account for the heterogeneity of data sources. To adjust for inter-survey differences in the socio-demographic composition among surveys, we standardized our outcome measures to reflect the fixed educational distribution of the population at the 2002 Russian Census: roughly the mid-point of the period over which our constituent studies were conducted. This will have also helped reduce variations due to selection bias in the recruitment of participants whereby survey participation tends to be higher among those who are most educated. However, this may have introduced a degree of underestimation of real population trends over time through the elimination of changes caused by a general rise of the educational level of the Russian population.

## Conclusion

Over the past 4 decades of observation, the prevalence of hypertension in Russia has been stable among men and women. Among women, this was due to two opposing trends: a decrease in mean systolic blood pressure and elevated blood pressure prevalence but an increase in the use of anti-hypertensives, potentially resulting in better control of raised blood pressure. In contrast, among men, there was little change in mean and elevated blood pressure and no improvement in its control. Despite improvements among women, the mean blood pressure (particularly diastolic at older ages) and the prevalence of elevated blood pressure remain high in Russia compared to England and the USA. Insufficient blood pressure control in Russia is an obstacle to further reduction of the CVD risks related to hypertension, especially among men.

## Supplementary Information

**Additional file 1: Supplementary Table S1.** Characteristics of surveys included in the analysis. **Supplementary Table S2.** Methods of blood pressure measurement. **Supplementary Table S3.** Mean Systolic Blood Pressure (mm Hg) by age, sex and survey adjusted for education. **Supplementary Table S4.** Mean Diastolic Blood Pressure (mm Hg) by age, sex, and survey adjusted for education. **Supplementary Table S5.** Percentage of individuals with elevated blood pressure (> = 140/90 mmHg) by age, sex, and survey adjusted for education. **Supplementary Table S6.** Percentage of individuals with hypertension (BP > =140/90 mmHg or use of medications) by age, sex, and survey adjusted for education.

**Additional file 2: Supplementary Figure S1.** Sex difference in the mean SBP in the age group 55–64 (SBP(m) minus SBP(w)) in Russian surveys (mm Hg). **Supplementary Figure S2.** Sex difference in the mean DBP in the age group 55–64 (DBP(m) minus DBP(f)) in Russian surveys (mm Hg). **Supplementary Figure S3.** Male to female odds ratio for the elevated blood pressure in the age group 55–64 in Russian surveys. **Supplementary Figure S4**. Male to female odds ratio for hypertension in the age group 55–64 in Russian surveys.

## Data Availability

The data that support the findings of this study are available from a variety of different sources (see Supplementary Table S1). The SAGE survey of the WHO is available at https://apps.who.int/healthinfo/systems/surveydata/index.php/catalog/68. The 2007–2008 and 2015–2016 NHANES are freely available for public use at https://www.cdc.gov/nchs/nhanes/index.htm. The 2009 and the 2016 HSE data can be accessed via the UK Data Service at https://beta.ukdataservice.ac.uk. Data extracts from LRC, WHO MONICA, Pitkäranta study 1992, 1997, 2002, 2006, Arkhangelsk study 2000, Monitoring of Arterial Hypertension, IFS 2, SAHR, ESSE and KYH were used under license for the current study and so are not publicly available. Researchers wishing to access any of these non-public datasets should contact the corresponding author who will provide contact details of the person responsible for any particular dataset(s).

## Abbreviations

CEE: Central and Eastern Europe
CVD: Cardiovascular diseases
DBP: Diastolic blood pressure
GBD: Global Burden of Diseases
HAPIEE: Health, Alcohol and Psychosocial Factors in Eastern Europe prospective cohort study
HSE: Health Survey for England
IFS: Izhevsk Family Study 2
IPCDR: International Project on Cardiovascular Disease in Russia
KYH: Know Your Heart study
LRC: Lipid Research Clinics Prevalence Study
MONICA: MONItoring trends and determinants in CArdiovascular disease study
NCD-RisC: Non-Communicable Disease Risk Factor Collaboration
OLS: Ordinary least squares
NHANES: National Health And Nutrition Examination Survey in the USA
Monitoring AH: Monitoring of Arterial Hypertension in the Russian Federation survey
SAHR: Stress Aging and Health in Russia; Lipid Research Clinics
SAGE: Study on Global AGEing and adult health
SBP: Systolic blood pressure
WHO: The World Health Organization
