# Supplementary material for: Long-term trends in blood pressure and hypertension in Russia: an analysis of data from 14 health surveys conducted in 1975–2017
Source: BMC Public Health. 2021 Dec 7;21:2226. doi: 10.1186/s12889-021-12320-4 (PMC8653591; doi:10.1186/s12889-021-12320-4)
Supplement: Supplementary file 1 — Additional file 1: Supplementary Table S1. Characteristics of surveys included in the analysis. Supplementary Table S2. Methods of blood pressure measurement. Supplementary Table S3. Mean Systolic Blood Pressure (mm Hg) by age, sex and survey adjusted for education. Supplementary Table S4. Mean Diastolic Blood Pressure (mm Hg) by age, sex, and survey adjusted for education. Supplementary Table S5. Percentage of individuals with elevated blood pressure (> = 140/90 mmHg) by age, sex, and survey adjusted for education. Supplementary Table S6. Percentage of individuals with hypertension (BP > =140/90 mmHg or use of medications) by age, sex, and survey adjusted for education. [file 12889_2021_12320_MOESM1_ESM.docx]

**Supplementary Table S1 – Characteristics of surveys included in the analysis**

| **Survey** | **Full name** | **Regions** | **Central year** | **Sample size** | **Age** | **Mean age** | **Share men (%)** | **Share high education (%)** | **Type of sample** |
| --- | --- | --- | --- | --- | --- | --- | --- | --- | --- |
| LRC/MONICA (1975-2002)*^, &^ | Lipid Research Clinics/WHO MONItoring trends and determinants in CArdiovascular disease [1-4] | Moscow, St. Petersburg | 1985 | 24410 | 18-82 | 46.0 | 74.8 | 41.5 | Seven random population-based samples from two typical districts in Moscow |
| Pitkäranta (1992)*^, &^ | Pitkäranta study [5,6] | Republic of Karelia | 1992 | 835 | 25-64 | 45.0 | 45.5 | 11.1 | Independent random samples from population register |
| Pitkäranta (1997) ^&^ | Pitkäranta study [5,6] | Republic of Karelia | 1997 | 749 | 25-64 | 44.5 | 41.3 | 13.1 | Independent random samples from population register |
| Arkhangelsk study (2000) | Arkhangelsk study [7] | Arkhangelsk | 2000 | 3660 | 18-94 | 42.9 | 53.3 | 20.9 | Random sample of residents registered in one outpatient polyclinic in Arkhangelsk |
| Pitkäranta (2002) ^&^ | Pitkäranta study [5,6] | Republic of Karelia | 2002 | 605 | 25-64 | 45.2 | 43.5 | 12.9 | Independent random samples from population register |
| Monitoring AH ( 2003-2004) | Monitoring of Arterial Hypertension [8,9] | 16 regions of Russia | 2004 | 30772 | 18-104 | 44.7 | 41.4 | 20.0 | Random samples of adults from selected 24 Russian regions recruited through outpatient polyclinics |
| Monitoring AH (2005-2008) | Monitoring of Arterial Hypertension [8,9] | 12 regions of Russia | 2006 | 31949 | 18-108 | 45.3 | 38.2 | 16.6 |  |
| Monitoring AH (2009-2010) | Monitoring of Arterial Hypertension [8,9] | 6 regions of Russia | 2010 | 11326 | 18-91 | 46.1 | 38.7 | 21.2 |  |
| Pitkäranta (2007) ^&^ | Pitkäranta study [6] | Republic of Karelia | 2007 | 483 | 25-64 | 46.8 | 39.8 | 16.8 | Independent random samples from population register |
| IFS 2 (2008-2009) | Izhevsk Family Study [10] | Izhevsk | 2008 | 1109 | 27-60 | 48.1 | 100.0 | 20.4 | Follow-up of random sample of men living in Izhevsk with additional participants of the same age |
| SAHR (2007-2009) ^&^ | Stress Aging and Health in Russia [11] | Moscow | 2008 | 1619 | 55-92 | 68.3 | 45.6 | 48.9 | Follow-up of a random sample of people aged 55+ from the seven  LRC/MONICA samples |
| SAGE (2007-2010)*^, &^ | WHO Study on global AGEing and adult health [12] | All Russia | 2008 | 2809 | 19-100 | 62.3 | 35.1 | 20.3 | Nationally representative sample of people aged 50+ with additional sample of younger people |
| ESSE (2012-2014) | Epidemiology of cardiovascular diseases in different regions of Russia [13] | 10 regions of Russia | 2013 | 22257 | 18-78 | 46.4 | 38.4 | 43.3 | Random samples of adults from selected  12 Russian regions recruited through outpatient polyclinics |
| KYH (2015-2017) | Know Your Heart study [14] | Arkhangelsk, Novosibirsk | 2016 | 5104 | 35-69 | 53.3 | 42.6 | 39.9 | Random sample of people from Arkhangelsk and Novosibirsk |
| HSE (2009) | Health Survey for England [15] | UK | 2009 | 4257 | 18-97 | 50.5 | 45.5 | 21.1 | Nationally representative sample |
| HSE (2016) | Health Survey for England [16] | UK | 2016 | 7826 | 18-90+ | n.a | 44.3 | 28.5 | Nationally representative sample |
| NHANES (2007-2008) | National Health And Nutrition Examination Survey [17] | USA | 2007 | 5789 | 18-80+ | 49.3 | 49.3 | 17.7 | Nationally representative sample |
| NHANES (2015-2016) | National Health And Nutrition Examination Survey [18] | USA | 2015 | 5992 | 18-80+ | 48.1 | 48.2 | 23.8 | Nationally representative sample |

*Notes.* * - survey’s data or tabulations are used by NCD-RisC : ^&^ - survey’s data or tabulations are used as the input source by GBD

**Supplementary Table S2 – Methods of blood pressure measurement**

| **Survey** | **Number of readings** | **Device** | **Type of device** |
| --- | --- | --- | --- |
| LRC/MONICA (1975-2002) | 2 | standard mercury sphygmomanometer | manual |
| Pitkäranta (1992) | 2 | standard mercury sphygmomanometer | manual |
| Pitkäranta (1997) | 2 | standard mercury sphygmomanometer | manual |
| Arkhangelsk study (2000) | 3 | DINAMAP R (Criticon) | semiautomatic |
| Pitkäranta (2002) | 2 | standard mercury sphygmomanometer | manual |
| Monitoring AH (2003-2004) | 2 | diaphragm standard mercury sphygmomanometer, phonendoscop for general practice IADM-OP manufactured by "Krasnogvardeec" | manual |
| Monitoring AH (2005-2008) | 2 | diaphragm standard mercury sphygmomanometer, phonendoscop for general practice IADM-OP manufactured by "Krasnogvardeec" | manual |
| Monitoring AH (2009-2010) | 2 | diaphragm standard mercury sphygmomanometer, phonendoscop for general practice IADM-OP manufactured by "Krasnogvardeec" | manual |
| Pitkäranta (2007) | 2 | standard mercury sphygmomanometer | manual |
| IFS 2 (2008-2009) | 3 | OMRON 705 IT | automatic |
| SAHR (2007-2009) | 2 | standard mercury sphygmomanometer | manual |
| SAGE (2007-2010) | 3 | OMRON R6 Wrist Blood Pressure Monitor, HEM-6000-E, Omron Healthcare Europe, Wegelaan 67-69 2312 JD Hoofddorp, The Netherlands. | automatic |
| ESSE (2012-2014) | 2 | OMRON М3 Expert | automatic |
| KYH (2015-2017) | 3 | OMRON 705 IT | automatic |
| HSE (2009) | 3 | OMRON HEM-907 | automatic |
| HSE (2016) | 3 | OMRON HEM-907 | automatic |
| NHANES (2007-2008) | 4 | a Calibrated® V-Lok® cuff, a Latex Inflation Bulb, and an Air-Flo® Control Valve. The pressure gauge is a Baumanometer® calibrated mercury true gravity wall model. | manual |
| NHANES (2015-2016) | 4 | a Calibrated® V-Lok® cuff, a Latex Inflation Bulb, and an Air-Flo® Control Valve. The pressure gauge is a Baumanometer® calibrated mercury true gravity wall model. | manual |

**Supplementary Table S3 - Mean Systolic Blood Pressure (mm Hg) by age, sex and survey adjusted for education**

| **Survey** | **Women** | | | | | | | **Men** | | | | | | |
| --- | --- | --- | --- | --- | --- | --- | --- | --- | --- | --- | --- | --- | --- | --- |
|  | **18-24** | **25-34** | **35-44** | **45-54** | **55-64** | **65-74** | **75 and older** | **18-24** | **25-34** | **35-44** | **45-54** | **55-64** | **65-74** | **75 and older** |
| LRC/MONICA, 1985 | 111.8(1.3) | 116.1(0.5) | 126.3(0.6) | 139.7(0.7) | 155.7(0.8) | 166.8(1.3) | - | 122.3(0.7) | 122.8(0.3) | 129.2(0.3) | 138.0(0.3) | 145.6(0.5) | 152.6(1.4) | - |
| Pitkaranta, 1992 | - | 123.7(1.2) | 136.3(1.8) | 156.0(2.4) | 170.4(2.7) | - | - | - | 132.1(1.8) | 138.8(1.7) | 150.1(2.5) | 157.5(2.9) | - | - |
| Pitkaranta, 1997 | - | 119.2(1.2) | 129.3(2.0) | 143.2(2.2) | 168.5(3.3) | - | - | - | 128.6(1.8) | 135.4(2.4) | 146.5(3.0) | 157.4(3.3) | - | - |
| Arkhangelsk study, 2000 | 114.7(0.6) | 117.9(0.7) | 123.9(0.8) | 136.5(1.1) | 148.9(1.5) | 156.4(2.0) | 154.3(3.2) | 121.2(0.6) | 128.2(0.6) | 130.8(0.7) | 139.0(0.8) | 147.2(1.4) | 156.6(1.8) | 150.9(4.1) |
| Pitkaranta, 2002 | - | 115.5(2.5) | 127.4(1.9) | 141.8(2.5) | 155.4(2.5) | - | - | - | 129.5(1.7) | 128.4(2.2) | 146.1(4.0) | 152.0(3.6) | - | - |
| Monitoring AH, 2003-2004 | 113.5(0.3) | 116.6(0.2) | 125.6(0.3) | 138.4(0.3) | 147.0(0.4) | 153.4(0.8) | 154.7(1.2) | 119.9(0.3) | 122.8(0.3) | 127.5(0.3) | 135.2(0.3) | 141.8(0.5) | 148.4(1.0) | 148.1(1.6) |
| Monitoring AH, 2005-2008 | 113.2(0.2) | 116.3(0.2) | 123.6(0.3) | 135.8(0.3) | 143.9(0.3) | 153.4(0.6) | 155.7(1.0) | 118.3(0.3) | 121.5(0.2) | 126.3(0.3) | 133.1(0.3) | 140.9(0.4) | 145.9(0.9) | 152.6(1.5) |
| Pitkaranta, 2007 | - | 118.6(1.7) | 124.3(2.1) | 140.1(2.4) | 153.1(2.6) | - | - | - | 125.5(2.1) | 133.6(2.8) | 149.4(4.8) | 152.2(3.7) | - | - |
| IFS 2, 2008-2009 | - | - | - | - | - | - | - | - | 135.1(0.9) | 139.4(0.8) | 147.6(0.7) | 153.4(1.6) | - | - |
| SAHR, 2007-2009 | - | - | - | - | 136.9(1.5) | 143.0(1.6) | 149.4(2.2) | - | - | - | - | 143.6(1.5) | 150.2(2.0) | 140.9(1.7) |
| SAGE, 2007-2010 | - | 119.0(1.5) | 122.3(1.4) | 132.9(0.8) | 141.6(0.8) | 147.6(0.8) | 151.4(1.0) | - | 123.7(1.4) | 126.3(2.2) | 132.4(1.0) | 138.5(0.9) | 146.1(1.2) | 143.5(1.3) |
| Monitoring AH, 2009-2010 | 114.1(0.4) | 117.9(0.4) | 124.8(0.5) | 136.0(0.4) | 143.0(0.4) | 145.6(0.9) | 151.3(1.7) | 118.8(0.5) | 121.8(0.4) | 127.6(0.5) | 133.7(0.5) | 140.5(0.6) | 143.6(1.3) | 145.5(2.0) |
| ESSE, 2012-2014 | 115.8(2.7) | 116.9(0.4) | 124.3(0.4) | 134.3(0.3) | 142.5(0.3) | 141.4(2.3) | - | 125.6(1.4) | 128.1(0.3) | 131.9(0.4) | 138.0(0.4) | 145.1(0.5) | 140.0(3.0) | - |
| KYH, 2015-2017 | - | - | 119.9(0.8) | 125.3(0.7) | 134.6(0.8) | 140.1(1.1) | - | - | - | 131.5(1.0) | 134.6(0.9) | 142.0(0.9) | 145.2(1.4) | - |
| HSE, 2009 | 114.1(0.9) | 114.1(0.8) | 117.1(0.7) | 124.5(0.9) | 132.4(1.1) | 136.7(1.2) | 139.7(1.5) | 127.3(0.9) | 128.2(1.1) | 127.7(0.8) | 130.8(0.9) | 136.4(1.1) | 135.8(1.2) | 136.2(1.7) |
| HSE, 2016 | 113.0(0.9) | 112.9(0.6) | 114.8(0.7) | 121.6(0.7) | 127.1(0.8) | 133.9(0.9) | 139.0(1.1) | 123.7(1.1) | 125.7(0.8) | 126.3(0.7) | 129.8(0.7) | 133.2(0.8) | 135.1(0.8) | 133.3(1.1) |
| NHANES, 2007-2008 | 109.1(0.5) | 110.7(0.6) | 114.3(0.6) | 121.2(0.8) | 130.0(0.9) | 134.7(1.2) | 141.2(1.3) | 117.2(0.6) | 118.8(0.5) | 121.3(0.6) | 125.1(0.7) | 129.6(0.9) | 132.8(1.0) | 134.7(1.1) |
| NHANES, 2015-2016 | 110.1(0.6) | 111.5(0.5) | 116.9(0.6) | 124.6(0.7) | 130.7(0.9) | 134.1(1.0) | 141.3(1.3) | 117.2(0.6) | 120.3(0.6) | 124.2(0.8) | 127.7(0.8) | 131.0(0.9) | 133.0(1.1) | 135.6(1.2) |

*Note: Standard errors are given in parentheses*

**Supplementary Table S4 - Mean Diastolic Blood Pressure (mm Hg) by age, sex, and survey adjusted for education**

| **Survey** | **Women** | | | | | | | **Men** | | | | | | |
| --- | --- | --- | --- | --- | --- | --- | --- | --- | --- | --- | --- | --- | --- | --- |
|  | **18-24** | **25-34** | **35-44** | **45-54** | **55-64** | **65-74** | **75 and older** | **18-24** | **25-34** | **35-44** | **45-54** | **55-64** | **65-74** | **75 and older** |
| LRC/MONICA, 1985 | 69.6(1.0) | 74.9(0.4) | 81.7(0.4) | 87.1(0.4) | 89.9(0.4) | 86.9(0.6) | - | 77.3(0.5) | 80.2(0.2) | 86.7(0.2) | 90.3(0.2) | 89.8(0.2) | 86.2(0.8) | - |
| Pitkaranta, 1992 | - | 72.4(1.0) | 82.2(1.1) | 86.5(1.1) | 91.0(1.2) | - | - | - | 76.0(1.6) | 84.2(1.3) | 86.3(1.3) | 87.4(1.4) | - | - |
| Pitkaranta, 1997 | - | 73.6(0.9) | 80.4(1.1) | 86.4(1.1) | 93.9(1.2) | - | - | - | 78.8(1.5) | 83.9(1.5) | 91.0(1.8) | 89.5(1.6) | - | - |
| Arkhangelsk study, 2000 | 72.4(0.5) | 74.8(0.6) | 79.6(0.6) | 85.2(0.6) | 87.8(0.9) | 89.3(1.1) | 89.5(1.8) | 59.2(0.5) | 70.2(0.6) | 77.4(0.6) | 83.4(0.6) | 84.4(0.8) | 86.5(0.9) | 81.2(2.0) |
| Pitkaranta, 2002 | - | 65.9(1.9) | 75.5(1.3) | 82.4(1.6) | 83.7(1.3) | - | - | - | 72.6(1.4) | 78.4(1.6) | 87.6(3.0) | 87.2(1.7) | - | - |
| Monitoring AH, 2003-2004 | 72.5(0.2) | 74.8(0.2) | 80.2(0.2) | 86.0(0.2) | 89.0(0.2) | 89.4(0.4) | 88.5(0.6) | 76.8(0.2) | 78.6(0.2) | 81.6(0.2) | 85.0(0.2) | 87.1(0.2) | 88.1(0.5) | 86.5(0.8) |
| Monitoring AH, 2005-2008 | 72.3(0.2) | 74.6(0.2) | 79.0(0.2) | 84.7(0.1) | 87.4(0.2) | 89.6(0.3) | 90.3(0.5) | 75.9(0.2) | 77.6(0.2) | 80.5(0.2) | 83.7(0.2) | 86.5(0.2) | 87.2(0.4) | 88.5(0.8) |
| Pitkaranta, 2007 | - | 71.5(1.5) | 78.0(1.3) | 84.6(1.1) | 88.0(1.3) | - | - | - | 77.5(1.7) | 86.5(1.9) | 91.2(2.7) | 89.8(1.7) | - | - |
| IFS 2, 2008-2009 | - | - | - | - | - | - | - | - | 83.3(0.7) | 88.6(0.6) | 91.4(0.4) | 92.7(0.8) | - | - |
| SAHR, 2007-2009 | - | - | - | - | 80.7(0.8) | 80.6(0.9) | 78.8(1.3) | - | - | - | - | 86.6(0.9) | 85.5(1.1) | 81.1(1.0) |
| SAGE, 2007-2010 | - | 77.2(1.2) | 80.0(1.0) | 85.2(0.5) | 88.1(0.5) | 89.0(0.5) | 89.6(0.6) | - | 79.1(1.2) | 81.5(1.7) | 84.5(1.6) | 87.8(0.6) | 88.8(0.7) | 85.9(0.9) |
| Monitoring AH, 2009-2010 | 72.3(0.4) | 75.3(0.3) | 79.3(0.3) | 84.2(0.2) | 86.8(0.2) | 87.9(0.6) | 89.7(1.0) | 75.3(0.4) | 77.6(0.3) | 81.0(0.3) | 83.8(0.3) | 85.7(0.3) | 86.3(0.8) | 87.2(1.1) |
| ESSE, 2012-2014 | 73.2(1.8) | 74.2(0.3) | 78.9(0.2) | 82.8(0.2) | 84.6(0.2) | 84.3(1.5) | - | 73.4(1.2) | 78.2(0.2) | 83.0(0.3) | 86.6(0.3) | 87.7(0.3) | 83.7(1.7) | - |
| KYH, 2015-2017 | - | 78.3(0.6) | 81.2(0.5) | 83.1(0.4) | 82.2(0.6) |  | - | - | - | 84.3(0.7) | 87.3(0.5) | 87.5(0.5) | 86.1(0.8) | - |
| HSE, 2009 | 67.6(0.8) | 70.3(0.6) | 72.6(0.6) | 76.9(0.6) | 76.5(0.6) | 74.0(0.7) | 69.2(0.8) | 67.9(0.9) | 72.9(0.8) | 75.4(0.7) | 78.5(0.6) | 78.2(0.7) | 73.8(0.7) | 68.0(1.1) |
| HSE, 2016 | 68.5(0.7) | 70.9(0.5) | 72.6(0.6) | 75.2(0.5) | 75.9(0.5) | 72.9(0.5) | 68.9(0.6) | 67.9(1.0) | 71.9(0.7) | 75.9(0.7) | 79.1(0.6) | 76.9(0.5) | 73.8(0.6) | 66.9(0.6) |
| NHANES, 2007-2008 | 64.3(0.5) | 67.4(0.5) | 71.4(0.5) | 73.0(0.5) | 71.6(0.5) | 67.7(0.6) | 64.1(0.7) | 64.4(0.6) | 70.1(0.5) | 75.8(0.5) | 77.0(0.5) | 75.3(0.5) | 70.4(0.6) | 65.4(0.7) |
| NHANES, 2015-2016 | 63.5(0.5) | 66.7(0.4) | 72.1(0.5) | 74.0(0.5) | 70.5(0.5) | 66.8(0.6) | 63.7(0.7) | 64.5(0.6) | 70.6(0.5) | 76.4(0.6) | 76.5(0.5) | 73.5(0.5) | 68.2(0.6) | 63.3(0.7) |

*Note: Standard errors are given in parentheses*

**Supplementary Table S5 - Percentage of individuals with elevated blood pressure (>=140/90 mm Hg) by age, sex, and survey adjusted for education**

| **Survey** | **Women** | | | | | | | **Men** | | | | | | |
| --- | --- | --- | --- | --- | --- | --- | --- | --- | --- | --- | --- | --- | --- | --- |
|  | **18-24** | **25-34** | **35-44** | **45-54** | **55-64** | **65-74** | **75 and older** | **18-24** | **25-34** | **35-44** | **45-54** | **55-64** | **65-74** | **75 and older** |
| LRC/MONICA, 1985 | 3.4(1.9) | 10.9(1.1) | 27.7(1.3) | 49.7(1.4) | 72.2(1.2) | 84.7(1.6) | - | 17.6(1.9) | 21.7(0.9) | 41.3(0.8) | 56.8(0.6) | 63.44(0.9) | 70.1(2.4) | - |
| Pitkaranta, 1992 | - | 10.6(3.0) | 34.5(4.2) | 69.4(4.5) | 87.4(3.5) | - | - | - | 31.7(6.2) | 44.5(5.6) | 60.9(5.0) | 70.2(4.9) | - | - |
| Pitkaranta, 1997 | - | 4.7(2.1) | 21.8(3.9) | 49.7(4.7) | 83.6(4.2) | - | - | - | 25.8(5.8) | 32.0(5.7) | 55.2(5.7) | 69.2(5.2) | - | - |
| Arkhangelsk study, 2000 | 0.4(0.4) | 3.3(1.1) | 13.9(1.8) | 38.3(2.4) | 60.3(3.4) | 70.7(3.9) | 57.1(5.9) | 3.9(0.9) | 16.6(2.1) | 25.1(2.2) | 45.1(2.4) | 61.7(3.3) | 76.4(3.3) | 66.3(8.0) |
| Pitkaranta, 2002 | - | 6.3(3.2) | 22.4(4.5) | 45.1(5.3) | 73.7(4.6) | - | - | - | 22.1(5.6) | 20.0(4.9) | 56.0(7.5) | 62.1(6.1) | - | - |
| Monitoring AH, 2003-2004 | 2.8(0.4) | 8.9(0.6) | 26.8(0.7) | 50.9(0.7) | 68.5(0.9) | 76.0(1.5) | 79.8(2.1) | 10.0(0.9) | 14.9(0.8) | 25.7(0.9) | 43.7(0.8) | 55.8(1.1) | 72.1(2.1) | 66.8(3.5) |
| Monitoring AH, 2005-2008 | 3.0(0.4) | 6.5(0.5) | 22.5(0.7) | 46.7(0.6) | 63.6(0.8) | 80.5(1.2) | 81.9(1.8) | 7.0(0.7) | 10.0(0.7) | 22.3(0.9) | 38.3(0.8) | 57.1(1.0) | 65.8(2.1) | 79.8(3.0) |
| Pitkaranta, 2007 | - | 7.4(3.5) | 14.8(4.5) | 47.7(5.6) | 66.7(5.1) | - | - | - | 6.5(4.4) | 36.3(8.1) | 54.4(6.9) | 69.9(6.3) | - | - |
| IFS 2, 2008-2009 | - | - | - | - | - | - | - | - | 35.9(3.0) | 48.7(2.3) | 63.9(1.5) | 70.9(2.7) | - | - |
| SAHR, 2007-2009 | - | - | - | - | 43.8(3.1) | 53.1(3.2) | 61.6(4.5) | - | - | - | - | 55.6(3.2) | 67.3(2.6) | 68.5(3.5) |
| SAGE, 2007-2010 | - | 13.7(4.3) | 17.7(3.8) | 41.3(2.2) | 59.4(1.8) | 68.9(1.7) | 74.7(1.9) | - | 8.9(4.2) | 25.0(6.0) | 37.3(2.7) | 52.7(2.3) | 63.3(2.6) | 66.6(3.1) |
| Monitoring AH, 2009-2010 | 3.1(0.8) | 7.7(0.9) | 23.4(1.3) | 49.3(1.1) | 65.6(1.1) | 75.4(2.3) | 77.3(3.8) | 7.4(1.2) | 10.9(1.1) | 23.5(1.6) | 40.7(1.4) | 58.7(1.5) | 70.7(3.5) | 76.9(5.3) |
| ESSE, 2012-2014 | 6.5(4.4) | 7.9(0.9) | 20.4(1.1) | 40.2(1.0) | 56.1(1.0) | 49.8(6.9) | - | 14.1(3.8) | 20.6(1.0) | 31.9(1.2) | 49.9(1.2) | 61.8(1.1) | 54.8(7.9) | - |
| KYH, 2015-2017 | - | - | 14.3(1.8) | 27.4(2.0) | 39.0(1.8) | 47.3(2.6) | - | - | - | 35.6(2.9) | 47.2(2.4) | 53.8(2.2) | 60.4(3.1) | - |
| HSE, 2009 | 0.9(0.9) | 3.8(1.3) | 9.5(1.6) | 18.9(2.3) | 32.3(2.8) | 43.7(3.2) | 47.6(3.5) | 7.3(2.5) | 19.7(3.2) | 18.0(2.3) | 25.3(2.7) | 42.0(3.1) | 38.6(3.1) | 45.9(4.3) |
| HSE, 2016 | 1.8(1.2) | 4.2(1.1) | 9.5(1.5) | 17.5(1.8) | 29.8(2.2) | 54.3(2.5) | 72.2(2.6) | 4.7(2.2) | 10.3(2.1) | 17.5(2.4) | 38.1(2.8) | 44.5(2.6) | 64.7(2.5) | 48.5(2.5) |
| NHANES, 2007-2008 | 0.9(0.5) | 3.6(0.9) | 6.5(1.1) | 16.0(1.7) | 29.4(2.1) | 35.1(2.5) | 48.2(2.7) | 4.8(1.1) | 5.6(1.1) | 14.2(1.7) | 22.3(1.9) | 27.3(2.1) | 33.1(2.5) | 37.5(2.7) |
| NHANES, 2015-2016 | 1.5(0.7) | 1.8(0.6) | 7.7(1.2) | 15.7(1.7) | 27.0(2.1) | 35.9(2.5) | 50.2(3.0) | 3.1(1.1) | 7.3(1.2) | 17.0(1.9) | 22.7(2.0) | 27.3(2.1) | 30.2(2.5) | 40.3(3.0) |

*Note: Standard errors are given in parentheses*

**Supplementary Table S6 - Percentage of individuals with hypertension (BP>=140/90 mm Hg or use of medications) by age, sex, and survey**

| **Survey** | **Women** | | | | | | | **Men** | | | | | | |
| --- | --- | --- | --- | --- | --- | --- | --- | --- | --- | --- | --- | --- | --- | --- |
|  | **18-24** | **25-34** | **35-44** | **45-54** | **55-64** | **65-74** | **75 and older** | **18-24** | **25-34** | **35-44** | **45-54** | **55-64** | **65-74** | **75 and older** |
| LRC/MONICA, 1985 | 3.5(1.9) | 12.0(1.2) | 31.2(1.4) | 52.4(1.4) | 74.4(1.2) | 86.7(1.5) | - | 19.1(2.0) | 22.4(0.9) | 41.9(0.8) | 57.9(0.6) | 65.0(0.9) | 70.4(2.4) | - |
| Pitkaranta, 1992 | - | 13.0(3.2) | 36.8(4.3) | 71.6(4.3) | 88.2(3.4) | - | - | - | 31.7(6.2) | 44.5(5.6) | 65.1(4.8) | 72.6(4.8) | - | - |
| Pitkaranta, 1997 | - | 10.8(3.0) | 24.3(4.0) | 54.2(4.7) | 84.4(4.1) | - | - | - | 27.0(5.8) | 33.1(5.8) | 56.4(5.6) | 71.3(5.0) | - | - |
| Arkhangelsk study, 2000 | 9.6(1.9) | 16.8(2.4) | 31.2(2.5) | 57.3(2.4) | 78.3(2.8) | 90.8(2.4) | 80.1(4.9) | 5.1(1.1) | 20.8(2.3) | 29.8(2.3) | 51.0(2.4) | 71.8(3.1) | 87.7(2.6) | 72.2(7.5) |
| Pitkaranta, 2002 | - | 7.6(3.4) | 31.8(4.9) | 50.4(5.3) | 78.2(4.3) | - | - | - | 22.1(5.6) | 21.3(5.0) | 62.4(7.4) | 63.5(6.1) | - | - |
| Monitoring AH, 2003-2004 | 3.9(0.5) | 11.1(0.6) | 32.9(0.8) | 59.3(0.6) | 76.1(0.8) | 84.2(1.3) | 84.7(1.9) | 11.8(0.9) | 17.0(0.8) | 28.4(0.9) | 48.0(0.8) | 62.2(1.1) | 78.6(1.9) | 72.8(3.3) |
| Monitoring AH, 2005-2008 | 7.9(0.6) | 12.9(0.6) | 30.6(0.8) | 56.8(0.6) | 74.3(0.7) | 89.3(0.9) | 93.3(1.2) | 12.4(0.9) | 14.2(0.8) | 28.2(1.0) | 46.4(0.9) | 67.5(1.0) | 77.8(1.9) | 86.9(2.5) |
| Pitkaranta, 2007 | - | 9.3(3.9) | 17.9(4.9) | 63.0(5.4) | 74.8(4.7) | - | - | - | 6.5(4.4) | 43.8(7.7) | 56.9(6.8) | 73.3(6.1) | - | - |
| IFS 2, 2008-2009 | - | - | - | - | - | - | - | - | 38.2(3.1) | 51.6(2.3) | 67.0(1.5) | 74.4(2.6) | - | - |
| SAHR, 2007-2009 | - | - | - | - | 60.0(3.4) | 73.9(2.6) | 76.8(3.5) | - | - | - | - | 66.6(3.0) | 79.0(3.1) | 79.0(3.0) |
| SAGE, 2007-2010 | - | 19.0(5.0) | 25.7(4.3) | 52.4(2.2) | 73.6(1.6) | 83.4(1.4) | 86.5(1.5) | - | 8.9(4.2) | 30.9(6.4) | 44.3(2.8) | 58.9(2.3) | 71.3(2.5) | 76.7(2.8) |
| Monitoring AH, 2009-2010 | 4.0(0.9) | 9.9(1.0) | 30.5(1.4) | 57.6(1.1) | 76.4(1.0) | 87.2(1.8) | 92.0(2.4) | 8.9(1.3) | 12.5(1.2) | 27.6(1.7) | 47.9(1.5) | 66.5(1.5) | 81.2(3.0) | 87.6(4.2) |
| ESSE, 2012-2014 | 15.5(6.0) | 13.0(0.9) | 30.7(1.1) | 56.1(0.8) | 75.3(0.7) | 80.5(4.6) | - | 16.9(4.1) | 24.6(1.1) | 38.2(1.3) | 59.5(1.1) | 72.4(1.0) | 69.5(7.3) | - |
| KYH, 2015-2017 | - | - | 23.4(2.2) | 45.5(2.2) | 72.6(1.6) | 84.4(1.8) | - | - | - | 43.7(3.0) | 58.3(2.4) | 71.9(1.9) | 82.8(2.4) | - |
| HSE, 2009 | 0.9(0.9) | 3.8(1.3) | 11.2(1.7) | 22.6(2.4) | 41.6(3.0) | 62.6(3.1) | 75.2(3.0) | 7.3(2.5) | 20.4(3.3) | 20.6(2.5) | 31.3(2.8) | 55.6(3.2) | 60.6(3.1) | 74.1(3.8) |
| HSE, 2016 | 1.7(1.2) | 4.3(1.1) | 12.2(1.7) | 22.8(2.0) | 38.5(2.3) | 57.1(2.4) | 69.3(2.6) | 4.4(2.2) | 9.9(2.1) | 19.5(2.5) | 36.0(2.7) | 48.7(2.6) | 64.7(2.5) | 64.0(2.8) |
| NHANES, 2007-2008 | 1.5(0.7) | 6.3(1.2) | 16.2(1.7) | 32.3(2.2) | 59.0(2.3) | 71.3(2.3) | 81.8(2.1) | 5.1(1.2) | 8.1(1.3) | 21.3(1.9) | 38.3(2.2) | 54.7(2.4) | 66.4(2.5) | 66.4(2.6) |
| NHANES, 2015-2016 | 1.8(0.7) | 4.0(0.9) | 19.2(1.8) | 32.1(2.1) | 59.2(2.3) | 72.9(2.3) | 79.7(2.3) | 3.7(1.0) | 8.6(1.3) | 23.7(2.1) | 40.0(2.3) | 58.9(2.3) | 62.4(2.5) | 69.6(2.7) |
| *Note: Standard errors are given in parentheses* | | | | | | | | | | | | | | |

**References**

1. Williams OD, Stinnett S, Chambless LE, Boyle KE, Bachorik PS , Albers JJ, Lippel K. Populations and methods for assessing dyslipoproteinemia and its correlates: the Lipid Research Clinics Program Prevalence Study. Circulation. 1986;73(Supplement I):I4-I11.
2. Deev AD, Konstantinov VV, Shestov DB. Population descriptions and methodology for US-USSR collaboration in Area 1 (Pathogenesis of Atherosclerosis) (Second prevalence study details for Moscow and Leningrad lipid research clinics). Atherosclerosis Reviews. 1988;17:103–9.
3. Deev AD, Oganov RG: Trends and determinants of cardiovascular mortality in the Soviet Union. Int J Epidemiol. 1989;18(Suppl 1):S137-S144.
4. Balanova YA, Shalnova SA, Deev AD, Konstantinov VV, Kapustina AV. Arterial hypertension prevalence in Moscow in different time intervals. Arterial’naya hypertensiya. 2013; 19(2):102-108.
5. Vlasoff T, Laatikainen T, Korpelainen V, Uhanov M, Pokusajeva S, Rogacheva A, et al. Ten year trends in chronic disease risk factors in the Republic of Karelia, Russia. Eur J Public Health. 2008;18:666–73.
6. Vlassof T, Laatikainen T, Korpelainen V, Uhanov M, Pokusajeva S, Tossavainen K, Vartiainen E, Puska P. Trends and educational differences in non-communicable disease risk factors in Pitkäranta, Russia, from 1992 to 2007. Scandinavian Journal of Public Health. 2015; 43: 91–98.
7. Averina M, Nilssen O, Brenn T, Brox J, Kalinin AG, Arkhipovsky VL. High cardiovascular mortality in Russia cannot be explained by classical risk factors. The Arkhangelsk study 2000. European Journ Epidemiol. 2003;18:871-78.
8. Oganov RG, Timofeeva TN, Koltunov IE, Konstantinov VV, Balanova YuA, Kapustina AV, et al. Arterial hypertension epidemiology in Russia: the results of 2003-2010 federal monitoring. Cardiovascular Therapy and Prevention (Russian Federation). 2011a; 10(1): 9 – 13.
9. Shalnova SA, Balanova YuA, Konstantinov VV, Timofeeva TN, Ivanov VM, Kapustina AV, Deev AD. Arterial hypertension: prevalence, awareness, treatment and control among Russian population. Russian cardilogical journal. 2006; 4(60):45-50.
10. Tomkins S, Collier T, Oralov A, Saburova L, McKee M, Shkolnikov V, Kiryanov N, Leon DA. Hazardous alcohol consumption is a major factor in male premature mortality in a typical Russian city: Prospective cohort study 2003–2009. PLoS One. 2012;7:e30274.
11. Shkolnikova MA, Shalnova SA, Shkolnikov VM, Metelskaya V, Deev AD, Andreev EM, Jdanov D, Vaupel JW. Biological mechanisms of disease and death in Moscow: Rationale and design of the survey on Stress Aging and Health in Russia (SAHR). BMC Public Health. 2009;9:293.
12. WHO Multi-country studies data archive. Russian Federation - Study on Global Ageing and Adult Health-2007/10, Wave 1. WHO. Report generated on October 24, 2013. <http://apps.who.int/healthinfo/systems/surveydata/index.php/ddibrowser/68/export/?format=pdf&generate=yes>. Accessed 17 February 2020.
13. Research Organizing Committee of the ESSE-RF project. Epidemiology of cardiovascular diseases in different regions of Russia (ESSE-RF). The rationale for and design of the study. Russian Journal of Preventive Medicine and Public Health = Profilakticheskaya meditsina. 2013;16(6): 25-34.
14. Cook S, Malyutina S, Kudryavtsev AV et al. Know your heart: Rationale, design and conduct of a cross-sectional study of cardiovascular structure, function and risk factors in 4500 men and women aged 35-69 years from two Russian cities, 2015-18. Wellcome Open Res. 2018;3:67.
15. University College London, Department of Epidemiology and Public Health, National Centre for Social Research. Health Survey for England, 2009. [data collection].3rd Edition. UK Data Service, 2015. Available from: <http://doi.org/10.5255/UKDA-SN-6732-2> Accessed 6 February 2020.
16. NatCen Social Research, University College London, Department of Epidemiology and Public Health. Health Survey for England, 2016. [data collection].3rd Edition. UK Data Service, 2019. Available from: [http://doi.org/10.5255/UKDA-SN-8334-3 Accessed 6 February 2020](http://doi.org/10.5255/UKDA-SN-8334-3%20Accessed%206%20February%202020).
17. Centers for Disease Control and Prevention (CDC). National Center for Health Statistics (NCHS). National Health and Nutrition Examination Survey Data. Hyattsville, MD: U.S. Department of Health and Human Services, Centers for Disease Control and Prevention, 2007. <https://wwwn.cdc.gov/nchs/nhanes/ContinuousNhanes/Default.aspx?BeginYear=2007> Accessed 6 February 2020.
18. Centers for Disease Control and Prevention (CDC). National Center for Health Statistics (NCHS). National Health and Nutrition Examination Survey Data. Hyattsville, MD: U.S. Department of Health and Human Services, Centers for Disease Control and Prevention, 2015. <https://wwwn.cdc.gov/nchs/nhanes/continuousnhanes/default.aspx?BeginYear=2015> Accessed 6 February 2020.
