# Supplementary material for: Long-term trends in blood pressure and hypertension in Russia: an analysis of data from 14 health surveys conducted in 1975–2017
Source: BMC Public Health. 2021 Dec 7;21:2226. doi: 10.1186/s12889-021-12320-4 (PMC8653591; doi:10.1186/s12889-021-12320-4)
Supplement: Supplementary file 2 — Additional file 2: Supplementary Figure S1. Sex difference in the mean SBP in the age group 55–64 (SBP(m) minus SBP(w)) in Russian surveys (mm Hg). Supplementary Figure S2. Sex difference in the mean DBP in the age group 55–64 (DBP(m) minus DBP(f)) in Russian surveys (mm Hg). Supplementary Figure S3. Male to female odds ratio for the elevated blood pressure in the age group 55–64 in Russian surveys. Supplementary Figure S4. Male to female odds ratio for hypertension in the age group 55–64 in Russian surveys. [file 12889_2021_12320_MOESM2_ESM.pdf]

Supplementary Figure S1 - Sex difference in the mean SBP in the age group 55-64 (SBP(m) minus SBP(w)) in Russian surveys (mm Hg)

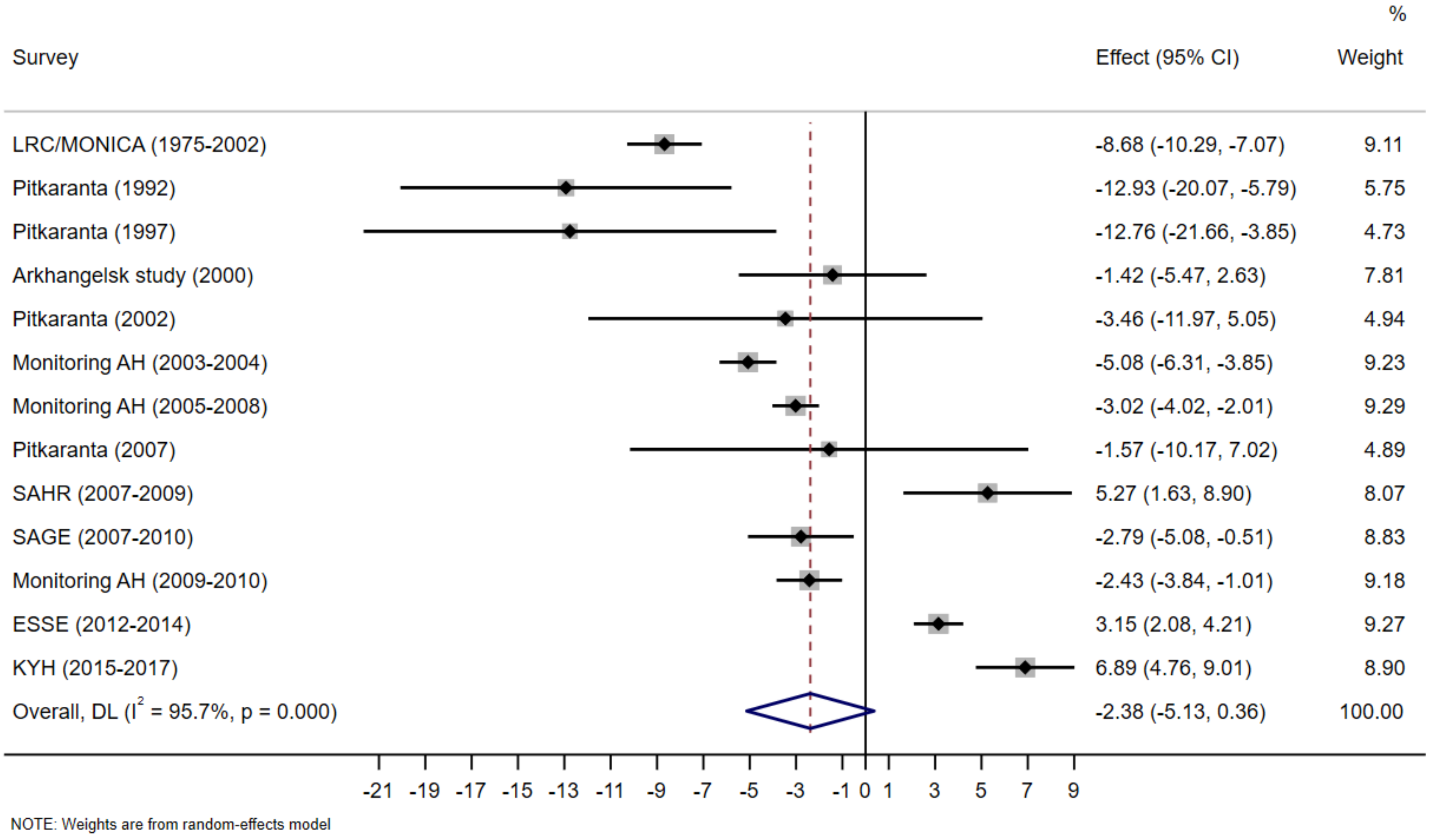

Note: results of random-effect meta-analysis with OLS regression linking SBP to sex adjusted for age and education

Supplementary Figure S2 - Sex difference in the mean DBP in the age group 55-64 (DBP(m) minus DBP(f)) in Russian surveys (mm Hg)

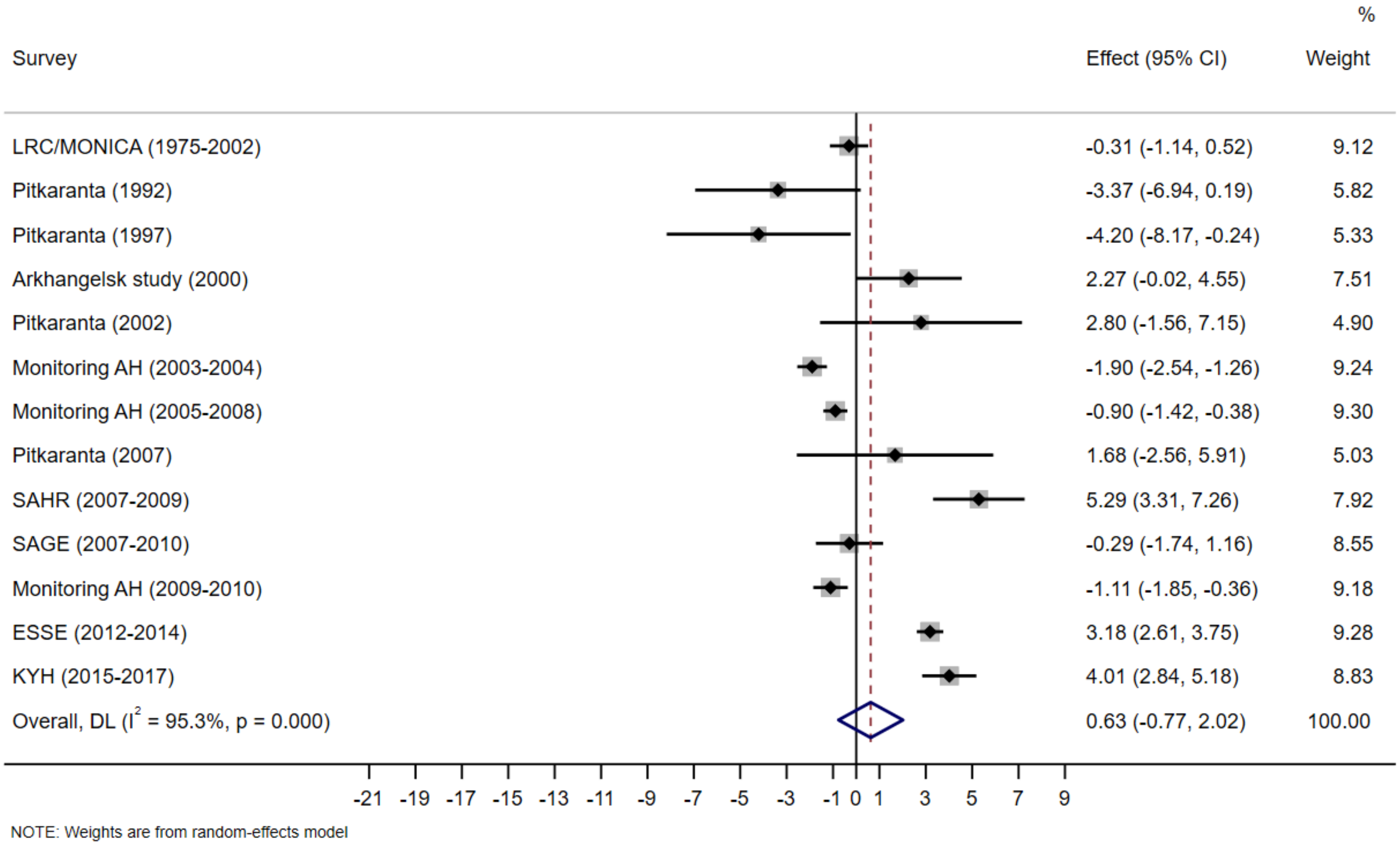

Note: results of random-effect meta-analysis with OLS regression linking DBP to sex adjusted for age and education

Supplementary Figure S3 – Male to female odds ratio for the elevated blood pressure in the age group 55-64 in Russian surveys

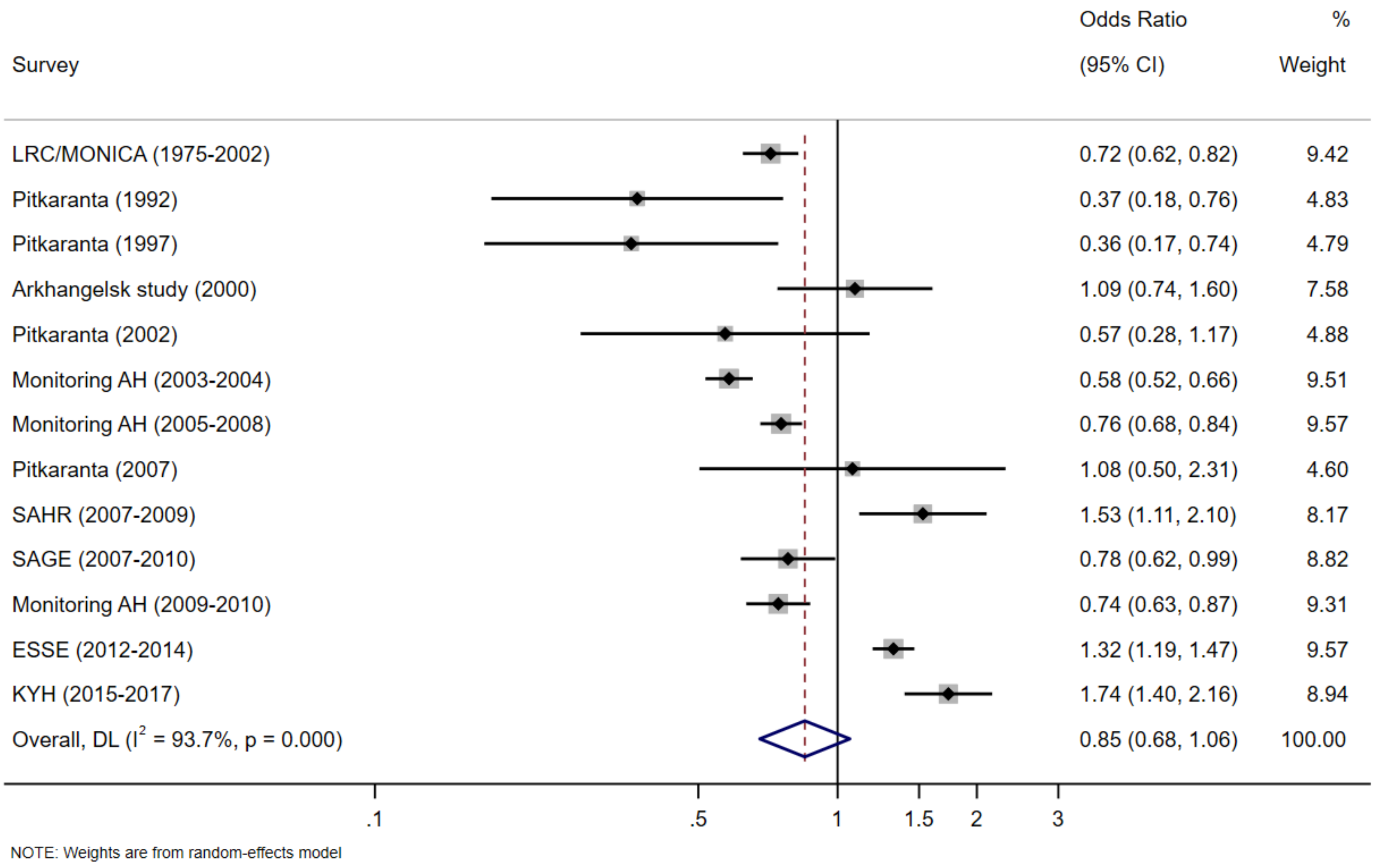

Note: random-effect meta-analysis with logistic regression linking odds of elevated BP to sex adjusted for age and education

Supplementary Figure S4 – Male to female odds ratio for hypertension in the age group 55-64 in Russian surveys

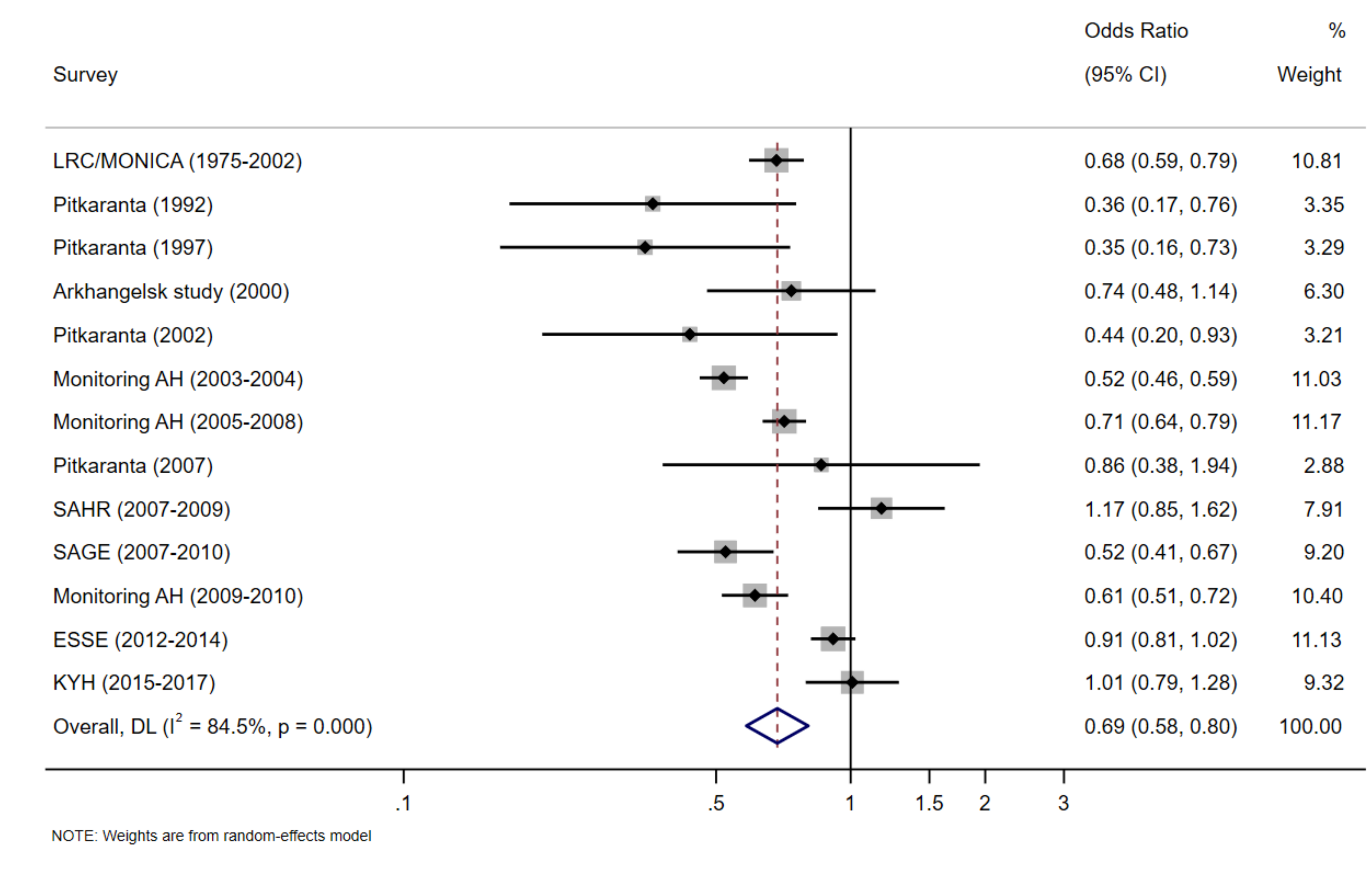

Note: results of random-effect meta-analysis with logistic regression linking odds of hypertension to sex adjusted for age and education
